# Effect of particle irregularity and particle size distribution on the morphology of packed beds of biochar particles

**DOI:** 10.1038/s41598-025-99495-7

**Published:** 2025-04-29

**Authors:** Zahra Ghasemi Monfared, J. Gunnar I. Hellström, Kentaro Umeki

**Affiliations:** Department of Engineering Sciences and Mathematics, Luleå University of Technology, Luleå, 971 87 Sweden

**Keywords:** X-Ray microtomography, Void fraction, Tortuosity, Pore network model, Dense graph, Particle size distribution, Energy science and technology, Porous materials

## Abstract

The heat and mass transfer in packed bed reactors (PBRs) are strongly influenced by the random packing of particles, making a thorough understanding of the packed bed structure crucial for optimal reactor design. This study investigates the impact of particle shape irregularities and size distributions on packing and transport properties using X-ray microtomography (XMT) imaging. Key morphological parameters, including void fraction and tortuosity, are extracted and analyzed. Two pore network models (PNMs)- one using cylindrical throats and another based on dense graph approach- are compared, with the dense graph model more accurately reflecting empirical tortuosity distributions. Results reveal that in monodispersed beds, void fraction decreases for particle diameters below 2 mm, nearing theoretical minimums for spherical packings, while tortuosity aligns with established models despite particle sphericity ranging between 0.6 and 0.8. In contrast, highly polydispersed beds exhibit lower void fractions compared to monodispersed beds, yet their tortuosity distributions remain similar. Visualization indicates small particles fill voids without blocking flow paths, preventing substantial tortuosity increases. These findings enhance understanding of packed bed behavior and provide valuable insights for designing biochar-based PBRs.

## Introduction

Packed bed reactors (PBRs) have numerous applications for a wide range of particle-based applications in the process and chemical industries, including catalytic reactors, gasifiers, energy storage systems, and metallurgical furnaces. PBRs feature porous beds that are randomly packed with particles whose structure could be described as porous materials^1^. One of the key characteristics of porous media within PBR that characterizes the inverse of its resistance to fluid flow through porous media is permeability^2^. The permeability of the porous media is determined by the Kozeny-Carman equation^3^ as follows:

1 $$k=\frac{{{\text{\varvec{\Phi}}}_{s}^{2}d_{p}^{2}}}{{180}}\frac{{{\varepsilon ^3}}}{{{{\left( {1 - \varepsilon } \right)}^2}}},$$

where $\varepsilon$ is the bed void fraction, *k* is the permeability, ${d_p}$ is the particle size, and ${\Phi _s}$ is the sphericity of the particles. Moreover, the diffusion of fluid within the pores of a packed bed defines as^4^:

2 $${D_{eff}}=\frac{\varepsilon }{\tau }/~\left( {\frac{1}{{{D_A}}}+\frac{1}{{{D_{Kn}}}}} \right),$$

where $\tau$ is the tortuosity of fluid flow through the gaps between the particles, ${D_A}$ is the molecular diffusivity of gas A in the mixture, ${D_{eff}}$ is the effective diffusivity, and ${D_{Kn}}$ is its Knudsen diffusivity.

It is crucial to have a thorough understanding of the packed bed morphology, including the tortuosity and void fraction, as suggested by Eqs. (1) and (2). The ratio of the void between the particles to the packed bed overall volume is known as the void fraction, which is a basic property of porous media. The size, shape, and packing configuration of the particles have a significant impact on the bed void fraction. Whereas irregularities in packed beds, such as disordered packings, polydispersed particles, and irregularly shaped particles show a greater range of bed void fractions^5^, the viable void fractions of the orderly packing of monodispersed spherical particles range from 0.26 to 0.46.

The other parameter is tortuosity, which is a measure of a porous material twisted or curved flow channels. Its definition is the ratio of the actual length of the path through the pores to the shortest straight-line distance between two points. In practical scenarios, the morphology of the pore network structure influences tortuosity in many cases^6–10^.

Four general forms of definitions of tortuosity can be found across different disciplines: geometrical, electrical, diffusive, and hydraulic tortuosity^7^. In particular, the current study focuses on geometrical tortuosity. Several theoretical approaches have been used to anticipate and analyze geometrical tortuosity, including the lattice percolation theory^8,11^ or the continuum percolation model combined with fractal theory in media with varying geometric shapes^12,13^.

Since Maxwell first established a correlation for electrical tortuosity in a porous media in 1873, there have been several studies attempting to create tortuosity equations that can account for a variety of packed beds^3,14–22^. These correlations were derived for packed beds of monodispersed spherical particles and primarily provide tortuosity as a function of the bed void fraction. It is unclear whether it is directly applicable and how accurate they are for polydispersed and irregular particle packings. Furthermore, there is still limited research on the geometric tortuosity of non-spherical particles with large packing fractions surrounding complicated porous structures. As a result, a thorough knowledge of how non-spherical particle properties, such as size, shape, and distribution, affect the pore space tortuosity is currently limited.

The pore network model with cylindrical throats is one method for estimating the tortuosity of packed beds. The PNM approach has become popular as a straightforward and effective method to model transport in porous materials^23–30^. In PNM, pores are represented as large void spaces within a porous medium, interconnected by narrower channels called throats. In contrast, the Lattice Boltzmann Method (LBM) simulates fluid flow at the microscale using the Boltzmann equation, accurately capturing complex pore geometries and providing a more detailed, though computationally intensive, estimation of tortuosity^8^. As an alternative to the widely used continuum modelling, which considers porous materials as volume-averaged continua without accounting for microscale details, PNM views the pore space inside the packed bed as a network of connected channels. Tortuosity is computed and flow path analysis is done using graph theory^31^. Using morphological simplifications of pore space geometry, the porous structure is commonly discretized into interconnected pores and straight cylindrical throats or channels^32^. In this study, we call this approach of using cylindrical throats as conventional PNM. As an alternative, the pore network can be obtained from a dense or skeletonized graph of pore space, which offers a more thorough description of the throats that connect pores and is similar to graph theory. It is another variant of the pore network model which, instead of limiting the throat pixels to a straight pipe-like line as seen in^33^, displays all of the throat pixels along connected pores. When skeletonized networks of pores and throats inside densely packed particle beds are employed instead of simplistic pore-throat models, the average tortuosity can be estimated with greater accuracy, as shown by Duan et al.^34^. However, based on the knowledge of the authors, no previous study has compared the accuracy of estimated tortuosity values obtained from conventional PNM (with cylindrical throats) and dense graph (with curved throats) approaches.

X-ray micro-tomography (XMT) is a highly helpful method for examining and deciphering the details of the packed beds porous structure^35–37^ and even further the reactivity of the packed beds of biochar particles^38,39^. The development of effective algorithms and increases in processing capacity have led to the emergence of strong tools for handling three-dimensional images produced by XMT. To derive tortuosity from tomographic data, for example, Cooper et al.^40^ and Al-Raoush et al.^41^ offered various open-source techniques.

This study investigates the tortuosity and void fraction of packed beds composed of irregularly shaped biomass-based char (biochar) particles with varying particle size distributions. X-ray microtomography (XMT) is used to capture three-dimensional images for further analyses of void fraction and tortuosity based on conventional pore network and dense graph models compared with available analytical models.

## Materials and methods

### Geometry and material properties

The studied packed beds were assembled in cylindrical containers with dimensions of 21 mm in diameter and 23 mm in height. The samples contain particles of densified biochar obtained from pyrolysis of dried spruce. Particle density of the biochar was 783 $\frac{{kg}}{{{m^3}}}.$ Both the production conditions for biochar and the sieving procedure are described in^42^.

In this experiment, six distinct samples with varying distributions and particle sizes ranging from 180 μm to 6.3 mm were used. This variation can demonstrate how the size distribution and particle size affect the morphology of the bed. Table 1 contains information on the logarithmic mean and standard deviation in the log-normal distribution of the particle size (volume-based distribution), as well as the minimum and maximum and mean particle sizes for each sample. Samples containing monodispersed particles have names beginning with MDP, while samples with polydispersed particles have names beginning with PDP. For the simplicity, we simply defined MDP as the particles that were obtained from narrow sieve classes while PDP as the particles from mixing two or more sieve classes. To further identify the degree of polydispersity, the coefficient of variation (*CV*) was used as a measure of dispersity in particle size distributions by relation, $\sqrt {{e^\sigma }^{2} - 1}$, where $\sigma$ is the logarithmic standard deviation of the particle size distribution^43^. *CV* < 0.3 represents moderate polydispersity and *CV* > 0.3 refers to high polydispersity where there is significant variability in particle sizes. Therefore, PDP1 and PDP2 are highly polydispersed and PDP3 is moderately polydispersed based on the data presented in Table 1.

**Table 1:** Characteristics of the particle size distribution of the samples used in the current study. (N.A.: not applicable)

Sample name	D_min_ [mm]	D_max_ [mm]	Median diameter ([mm])	Logarithmic mean [-]	Logarithmic standard deviation [-]	CV
MDP1	0.315	0.4	0.357	N.A.	N.A.	N.A.
MDP2	2	3.15	2.575	N.A.	N.A.	N.A.
MDP3	4	6.3	5.15	N.A.	N.A.	N.A.
PDP1	0.18	3.15	1.665	– 0.8	1.42	2.55
PDP2	0.18	5	2.59	– 0.48	1.71	4.20
PDP3	3.15	6.3	4.725	1.29	0.32	0.33

### Utilizing X-ray micro-tomography (XMT)

**Fig. 1:**
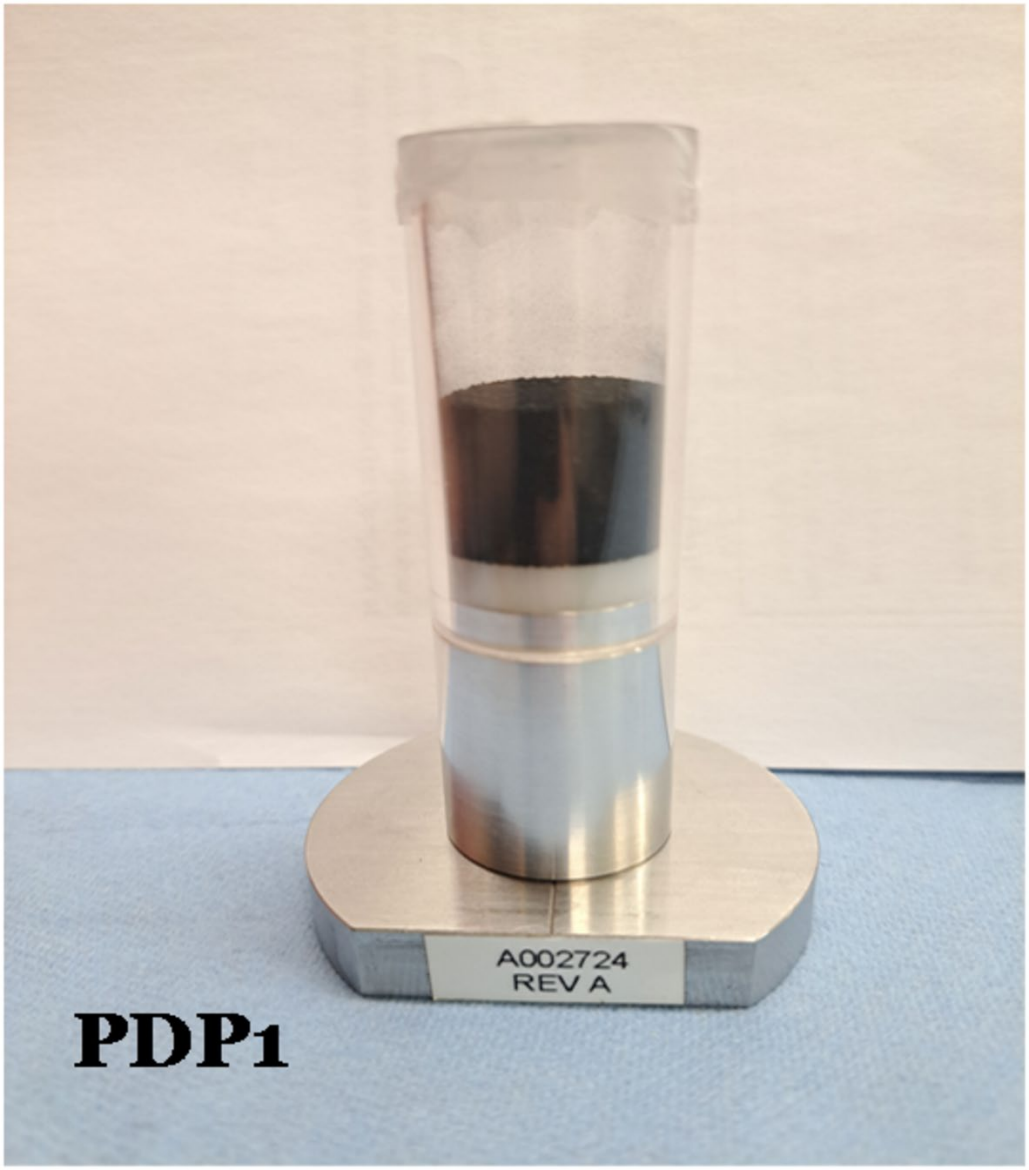
The sample holder and the packed bed of PDP1 were used for XMT.

As seen in Fig. 1, the samples have been handled to obtain the structure of the packed beds. The sample container was loaded with char particles to a height of approximately 23 mm, and the packed bed was gently tapped ten times (following the European Biochar Certificate’s recommendation^44^) to bring the bed packing densities in line with those reported in^42^.

After that, the samples were placed inside the Zeiss Xradia 510 Versa, as an XMT device. The X-ray beam is directed at the material within the XMT. The X-ray beams are either absorbed by the material, diffracted, or transmitted through it^45^. The detectors then pick up the transmitted radiation that has less intensity since it has passed through the sample.

A 3 W tube power using LE1 (low-energy X-ray 1) or LE2 X-ray filter (depending on the sample features) has been employed to reduce beam hardening artifacts. The scanning has been done with an X-ray tube voltage in the range of 40 kV, depending on the particle size distribution.

In order to reduce artifacts in reconstruction, a complete 360° rotation of 1601 projections has been collected for every scan. Depending on the feature size of each sample, different resolutions and acquisition periods were used to obtain the images. The resolutions of 5 μm have been chosen for MDP1 and PDP2, whose smallest particle sizes are equal to 0.315 mm and 0.18 mm, respectively, and 22 μm for the remaining samples with larger particle sizes. In XMT imaging, at least 3–5 voxels per feature are recommended for accurate geometrical reconstructions^46^. The chosen resolution in this study meets this criterion, reducing the risk of staircasing effects, surface roughness artifacts, and shape distortions.

**Fig. 2:**
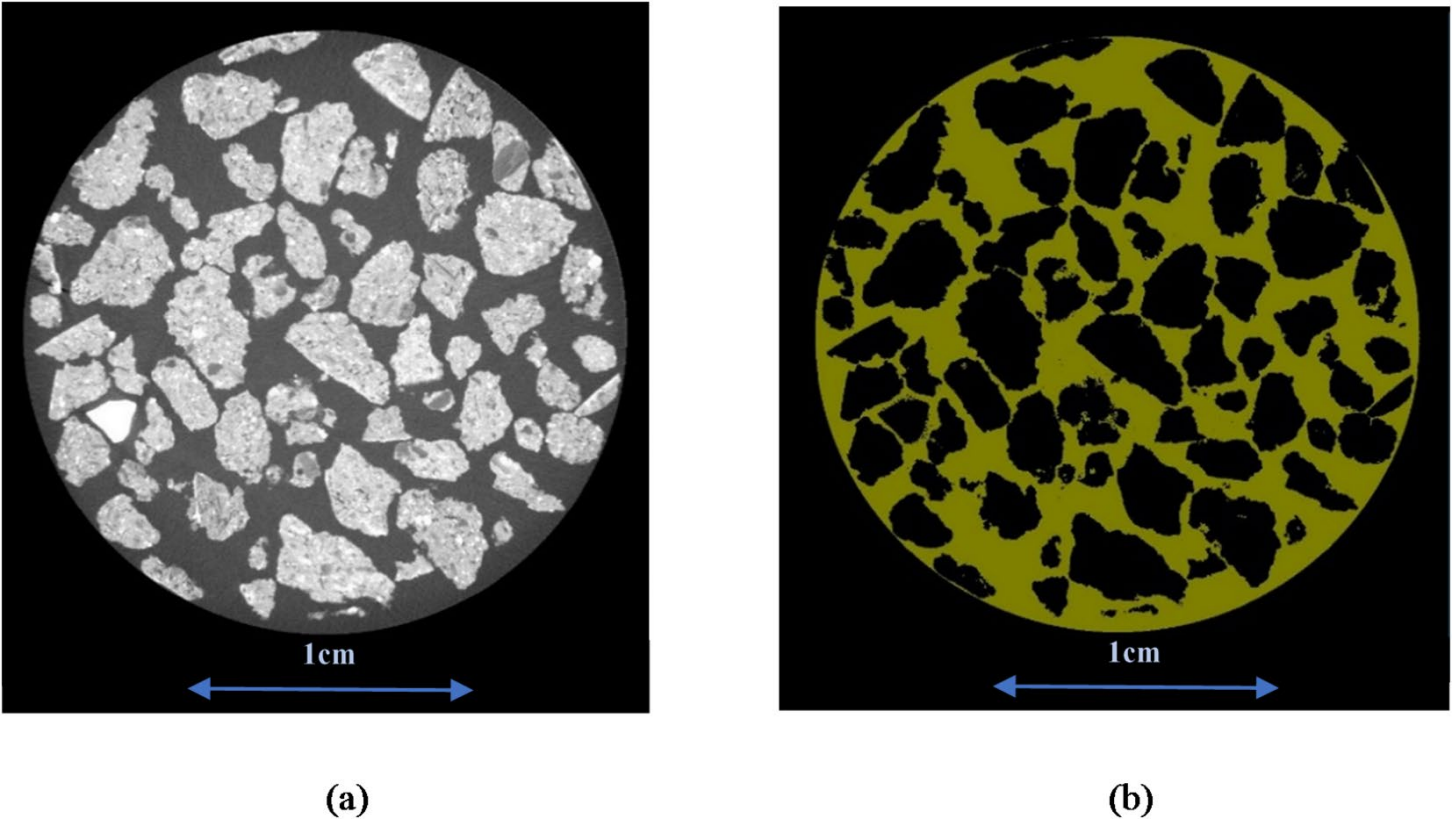
(a) A sample of the raw grayscale image for MDP2 was obtained from the XMT. (b) The segmented image of MDP2 sample binarized into particles and void space between them.

The DRAGONFLY® software (Version 2022.2 Build 1367) receives the raw data export. A stack of 16-bit TIFF images was the result of this technique. An example of an X-ray raw grayscale image of the horizontal plane from the sample MDP2 is shown in Fig. 2a.

As seen in Fig. 2a, the XMT settings have produced sufficient contrast in the region between the biochar particles and the void space interface, where the darker regions represent void space, and the lighter regions correspond to the solid phase.

A 3D depiction of the pore space structure was generated by stacking the binarized images. Binarized images were generated by segmenting each stack of grayscale images. A thresholding approach involves manual interaction to select appropriate thresholds from the bimodal histogram of grayscale intensity values so that the particles and the vacant space are identified with accuracy. The density differences of various phases inside the material were represented by their grayscale appearance, which correlates to X-ray attenuation. As shown in Fig. 2b, the binarized image captured the details of the particle morphology accurately, with the yellow regions representing void space and the black regions within the domain corresponding to the solid phase.

### Calculation of void fraction

#### Analytical values for the void fraction of particle packings

A fundamental aspect in characterizing the morphology of packed beds is the bed void fraction, representing the proportion of void space volume between the particles to the total volume of the packed bed. In this paper, the internal pore of the particles is not regarded as void. Sphere packing serves as a reference point for understanding the effect of particle irregularity and size distribution on void fraction. Analytical investigations into sphere packing encompass the exploration of various configurations, including face-centered cubic (FCC) and body-centered cubic (BCC) arrangements. Each packing arrangement manifests unique void spaces between the spheres. For example, the FCC arrangement, with spheres positioned at the vertices of a cube and on its faces, achieves the maximum achievable packing fraction for spherical particles, resulting in a void fraction of 0.26. Conversely, the BCC configuration yields a void fraction of 0.32. These void fraction values can be analytically obtained for specifically defined arrangements of mono-sized spherical particles. They serve as reference points for comparing results obtained through XMT analysis.

#### Calculation of void fraction from XMT-based images

To extract the void fraction from XMT images, slice analysis was conducted with the software DRAGONFLY^®^ on vertical slices, which essentially represent cross-sectional planes parallel to the bottom of the bed. Figure 2b illustrates a schematic of the segmented image from the same slice of MDP2 as depicted in Fig. 2a. In Fig. 2b, the yellow regions represent the void spaces, while the black spots denote the particles that have been binarized during the segmentation process.

The area-averaged void fraction can then be obtained for each section as below:

3 $${\bar {\varepsilon }_{section}}=\frac{{{A_{void}}}}{{{A_{section}}}},$$

where ${A_{section}}$ is each circular section’s area and ${A_{void}}$ is each section’s area of void space. ${A_{void}}$ can be determined through slice analysis in DRAGONFLY^®^ for slices taken at longitudinal intervals based on previously mentioned resolutions. The area-based void fractions from all sections are then averaged to obtain the overall bed void fraction for each sample.

### Calculation of tortuosity

In this study, geometrical tortuosity was calculated using the 3D XMT data. In the pore network models (conventional PNM and dense graph), pores are conceptualized as the larger void spaces within a porous medium, connected by narrower channels known as throats between pores, and the tortuosity was computed as the ratio of the total pathway length, each of which measured from the center of one pore to the center of another pore through the connecting throats, to the straight-line distance between the inlet and the outlet.

#### Calculation of tortuosity based on the conventional pore network model (PNM)

With the 3D pore structure of the packed bed obtained from XMT data, statistical information on the tortuosity of the packed bed can be extracted. PNM discretizes the porous structure into interconnected pores and throats, forming a network that represents the pore-scale geometry of the material. In the current study, the algorithm offered by OpenPNM package in Dragonfly software has been implemented for extraction of the conventional pore-throat network of the packed beds. This algorithm identifies pores as local maxima of an inscribed sphere map, while throats are defined as the narrowest cross-sections where these spheres meet. This approach ensures that pores represent the largest void spaces, and throats are the critical constrictions controlling transport properties. (see Ref^31^. for additional information about the algorithm).

**Fig. 3:**
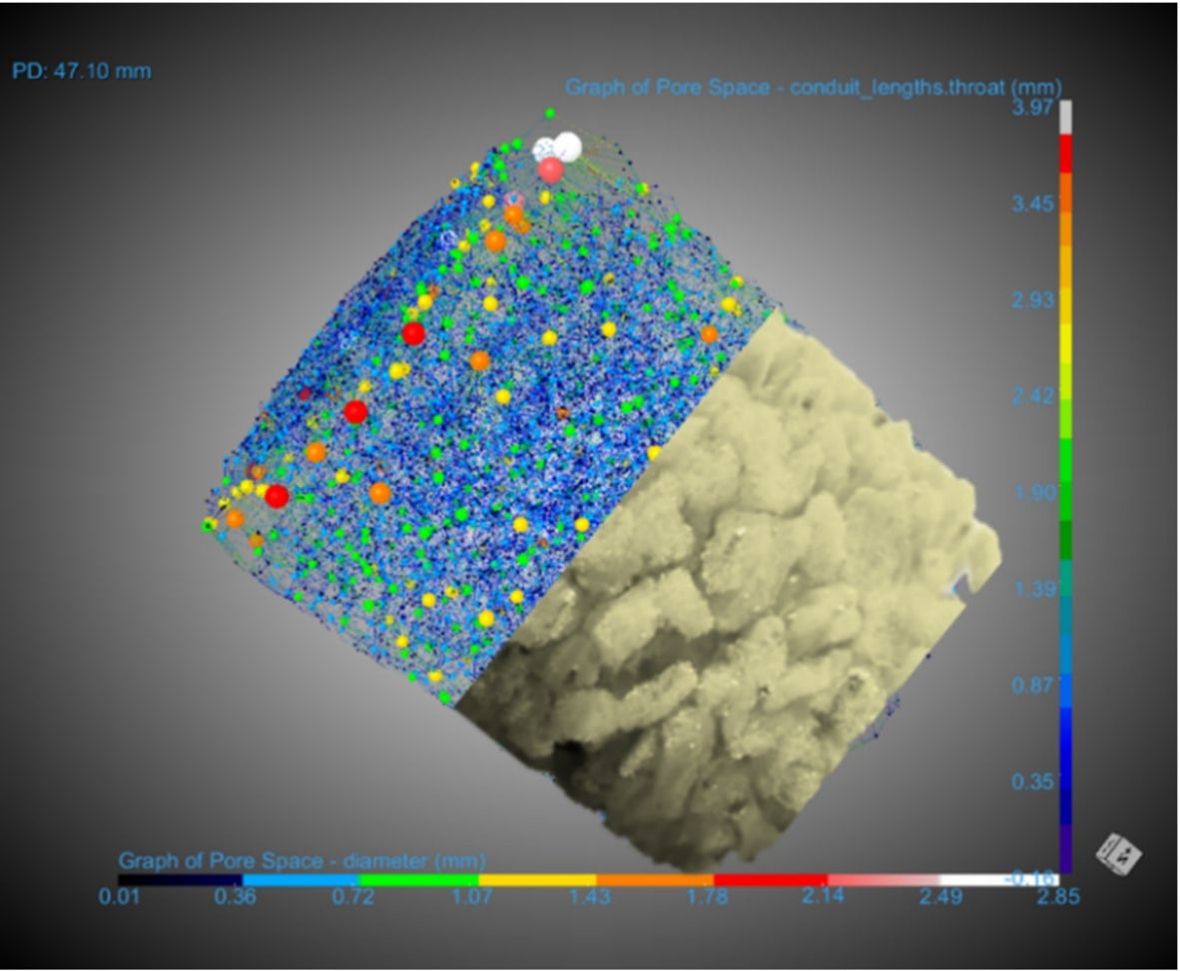
The pore-throat network extracted from the pore structure of the MDP2. The 3D image was generated using DRAGONFLY 2022.2 (software available at https://www.theobjects.com/dragonfly)^47^.

A schematic of the pore-throat network within the MDP2 sample is shown in Fig. 3. The bottom half depicts the 3D image of the packed bed, while the top half illustrates the extracted pore-throat network. It can be observed that the pore diameters range from 0.01 to 2.85 mm, and the lengths of the channels are mostly below 0.35 mm, except for regions near the walls, where the lengths can reach up to approximately 3.45 mm.

**Fig. 4:**
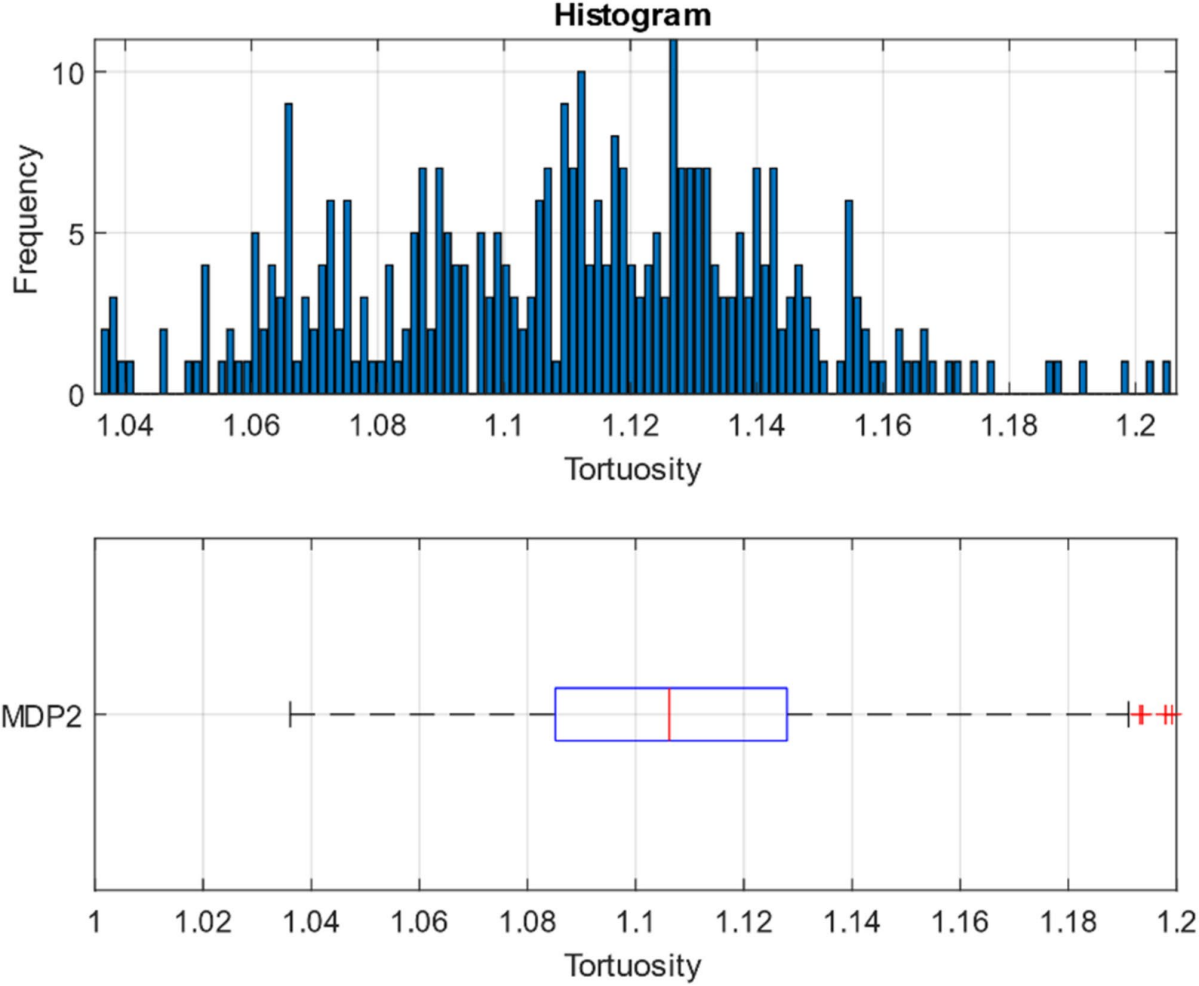
Top) the histogram of the tortuosity values in the mdp2 packed bed from the inlet to the outlet. Bottom) the box chart related to the tortuosity values.

After obtaining the pore-throat network, all possible pathways were analyzed under the constraint that the flow direction is aligned with the height of the cylinder, with the inlet at the bottom and the outlet at the top. The result is a histogram of tortuosity values computed through nearly all possible paths from the inlet to the outlet. An example of the OpenPNM model outcome for MDP2 is displayed in Fig. 4.

The top image in Fig. 4 depicts the histogram of tortuosity values distribution. For sample MDP2, the smallest values correspond to the paths that offer the least resistance to fluid flow, typically found near the walls. The largest values in the histogram represent the longest possible connections from the inlet to the outlet, which contribute to the average tortuosity of the bed but may be physically impractical for fluid passage due to constraints on throat diameter and channel lengths.

The bottom image in Fig. 4 shows a box chart of the tortuosity distribution for MDP2. In the box chart, the leftmost line represents the minimum tortuosity observed, which is 1.03. The left side of the rectangle indicates the first quartile (the lower 25% of the data), the middle line represents the median, and the right side of the rectangle represents the third quartile (higher than 75% of the data but lower than the remaining 25%). The rightmost line indicates the maximum tortuosity value, approximately 1.18. The red cross-like markings at the end of the range (~ 1.2) indicate the outlier values where the data points exceed beyond the third quartile (or below the first quartile). These values show that some tortuosity measurements extend beyond the expected range but still exist within the dataset.

#### Calculation of tortuosity based on the dense graph approach

As discussed earlier, to analyze the pore-throat networks within porous packed beds, one could employ dense graph representation of the pore space skeleton, followed by the computation of tortuosity from this network. This representation conceptualizes the structure of the porous packed bed as a graph, with nodes representing pores and edges denoting connections between them. Scalar values, such as edge lengths or tortuosity distributions, can then be derived from this graph. In the current study, the dense graphs of the pore structure of the samples have been obtained using the relevant DRAGONFLY® module.

**Fig. 5:**
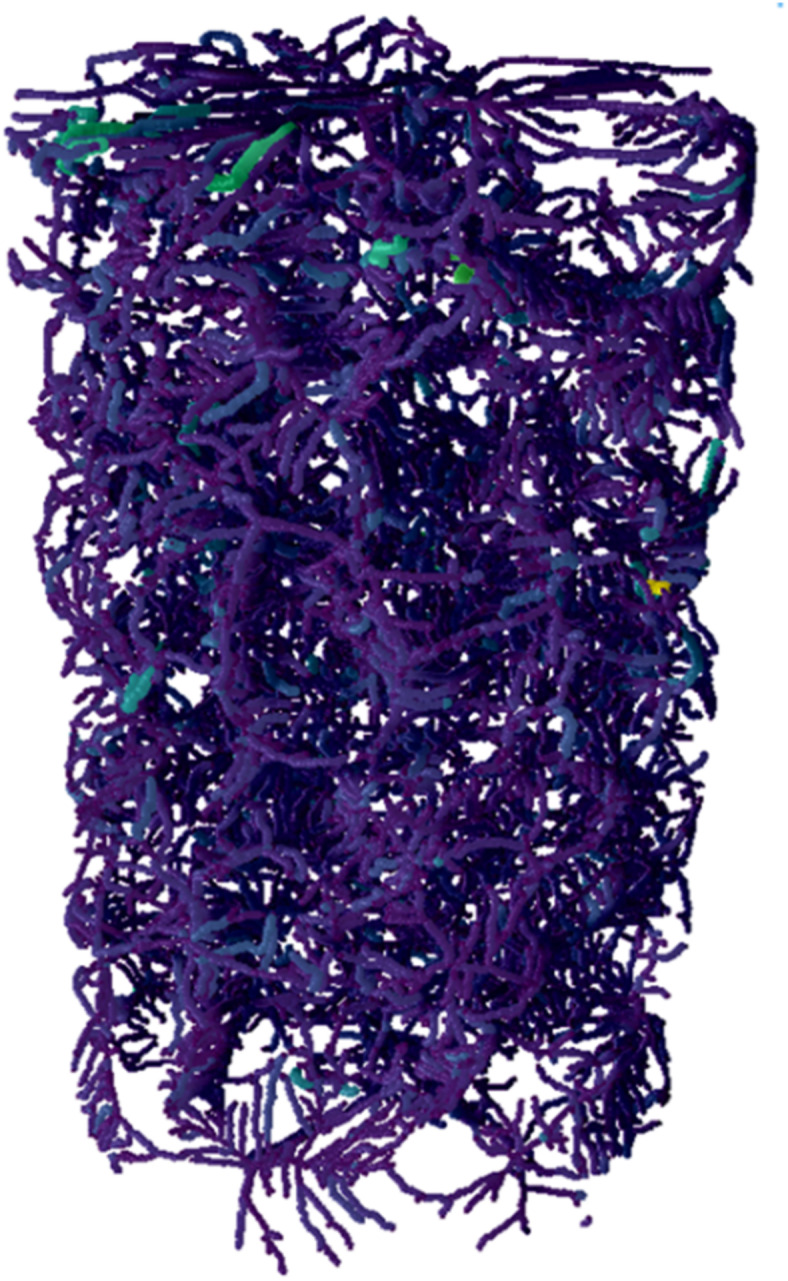
The pore-throat network obtained by the dense graph method for MDP2. The 3D image was generated using DRAGONFLY 2022.2 (software available at https://www.theobjects.com/dragonfly)^47^.

Once the dense graph is extracted, tortuous paths from the input to the output are identified and measured, and tortuosity is computed for each path^48^. This process involves calculating all potential tortuous pathways, ensuring alignment of flow direction with the cylinder height, with the inlet at the bottom and the outlet at the top. Consequently, a histogram of tortuosity values is generated similar to the conventional PNM method, computed across nearly all conceivable routes from the inlet to the outlet. It is important to note that both models use the same nodes (pores) based on the real 3D geometry, but the difference lies in how the pathways (throats) between pores are represented. In the conventional PNM, the transport pathways are simplified as straight lines, disregarding the naturally curved or complex trajectories found in real porous media. However, the pathways between pores are influenced by the surface curvature of the particles. In contrast, the dense graph representation captures the intricate connectivity and curved pathways between pores, offering a more accurate depiction of the porous structure. This leads to a more realistic estimation of tortuosity, as the dense graph model accounts for the actual path lengths and complexities of the pore network. An example of the pore-throat network extracted by the dense graph approach (MDP2 sample) is shown in Fig. 5. As it can be seen, the throats connecting the pores are curved and as mentioned before, they cover all the pixels of the throat between two pores.

There are previous researches that studied the effect of the sample holder diameter on mono spherical particles (e.g., Ref^49^) for estimation of void fraction, however, these studies may not be applicable to tortuosity calculation of polydispersed and nonspherical particles. To ensure that the obtained tortuosity distribution is not sensitive to radial dimensions and the size of the sample holder is large enough for the samples with larger particles, the sensitivity of the results to the size ratio has been checked for PDP3. To do so, sub-domains with different diameters starting from the center of the cylinder were created. It is important to note that if the sample holder is large enough for larger particles, the results of tortuosity calculations would be also not sensitive for samples with smaller particles.

**Fig. 6:**
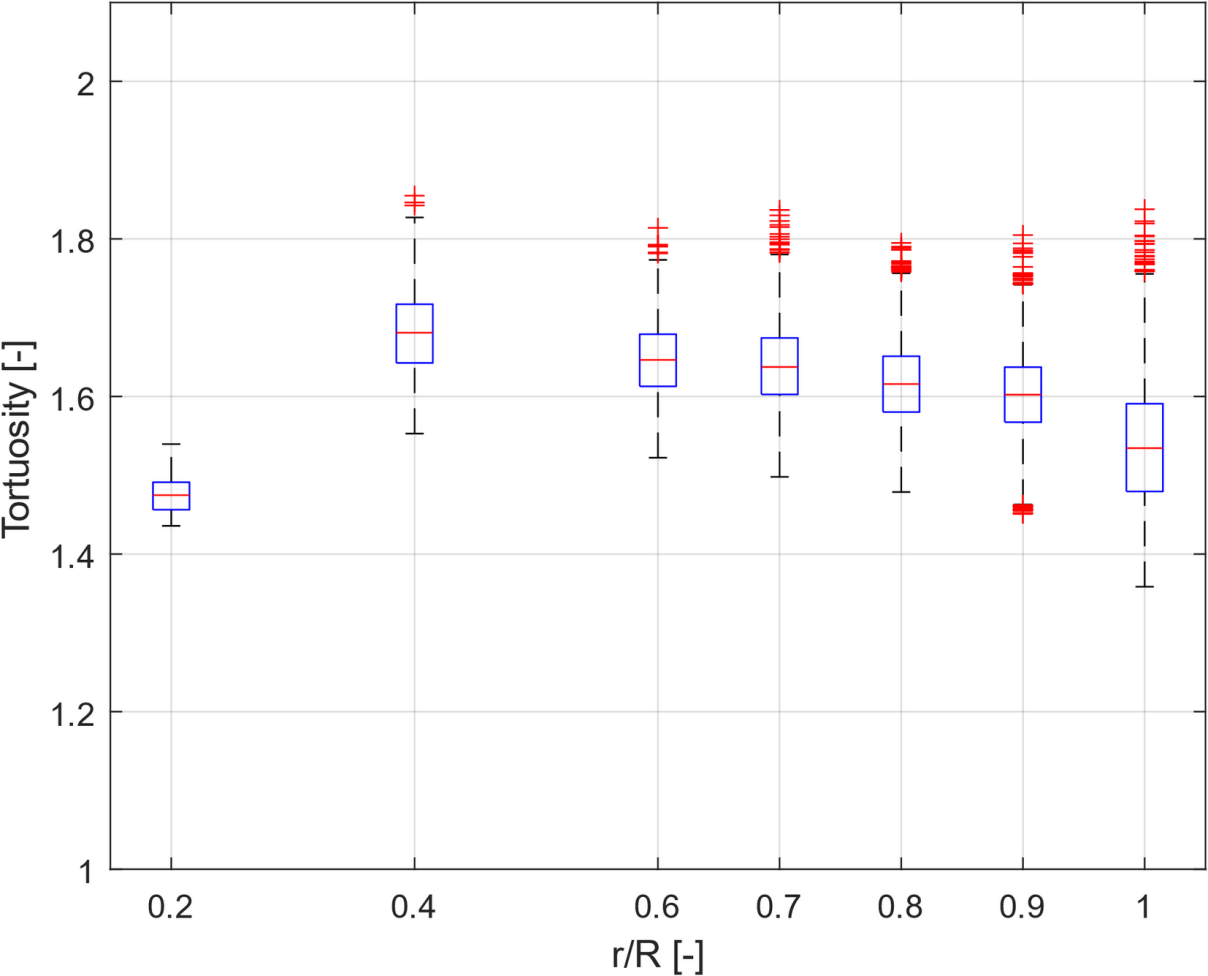
The tortuosity distribution within different subdomains as a function of the normalized subdomain radius (r/R) for sample PDP3.

The tortuosity distribution within the subdomains of PDP3 sample is presented in Fig. 6 as a function of the normalized subdomain radius (*r/R*), where *r* is the radius of the subdomain and *R* is the radius of the cylinder (sample holder). As expected, values near the wall were different due to the wall effect on void fraction. Changes in the subdomain size for the ratios ranging from 0.8 to 0.9, did not lead to significant changes in tortuosity values (less than 1% difference). Therefore, for higher accuracy, further analysis of tortuosity distributions within all the samples have been carried out within the subdomains with radii of 0.9*R*.

## Results and discussions

### Void fraction

#### Monodispersed samples

The void fraction of a packed bed is influenced by the shape and size of individual particles as well as the size distribution.

**Fig. 7:**
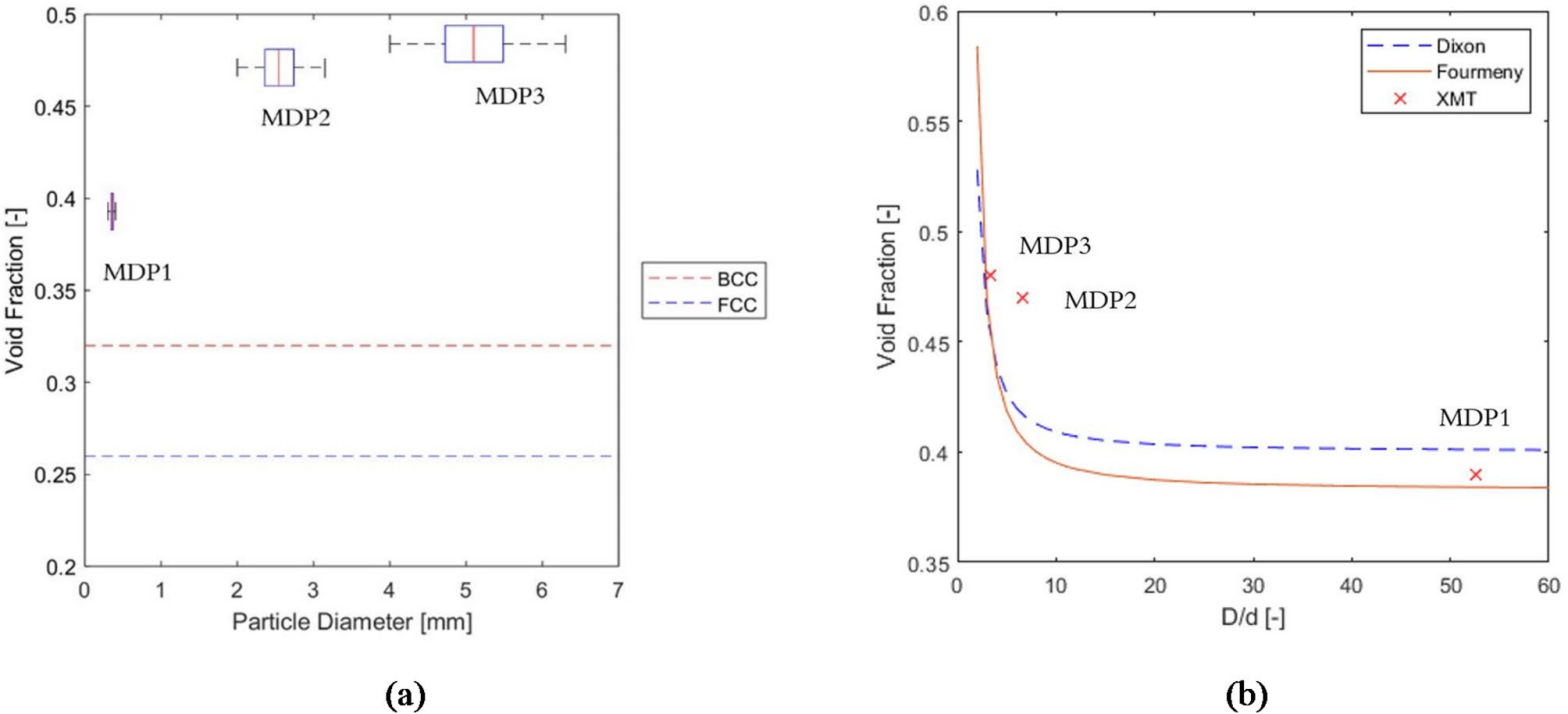
(a) Comparison between the void fractions of samples MDP1, MDP2, and MDP3, and the analytical void fractions for BCC and FCC packing arrangements. ((b) The effect of D/d ratio on void fraction with respect to the correlations by Dixon^49^ and Foumeny et al.^51^.

Figure 7 illustrates the relationship between void fraction and (a) particle size as well as (b) tube-to-particle diameter ratio (D/d) for three samples, MDP1, MDP2, and MDP3, with size specifications detailed in Table 1. In monodispersed packed beds it shows an increase in void fraction when the particle diameter is larger than 1/10 of tube diameters^50^. In addition, for irregular particles, large particles lead to larger gaps between the particles and, consequently, higher values of void fraction^51^. As particle size increased (from an average size of 0.575 mm in MDP1 to 5.15 mm in MDP3), the void fraction also increased, diverging further from the maximum analytical value for tightly packed, mono-sized spherical particles in BCC arrangements (0.32).

Figure 7b compares the effect of D/d ratio on void fraction with correlations by Dixon^50^ and Foumeny et al.^52^. The general trend of higher void fraction at lower D/d ratio agrees with the literature, but the measurement data did not show the exponential increase as previous studies. Although it is not possible to exclude the conventional effect of tube-to-particle diameter ratio, the data indicates the existence of additional effects.

This deviation can also be attributed to the irregular shapes^51^ if the particles are not in spherical forms. Therefore, sphericity variation within each sample, indicating 1 for perfect sphere and ranging from 0 to 1 for irregular shapes, was analysed. As per definition, the sphericity of particles is defined as,

4 $$Sphericity=~\frac{{{{\left( {6{\pi ^{\frac{1}{2}}}{V_p}} \right)}^{\frac{2}{3}}}}}{{{A_p}}},$$

where *V*_*p*_ and *A*_*p*_ are the volume and surface area of the particle, respectively.

**Fig. 8:**
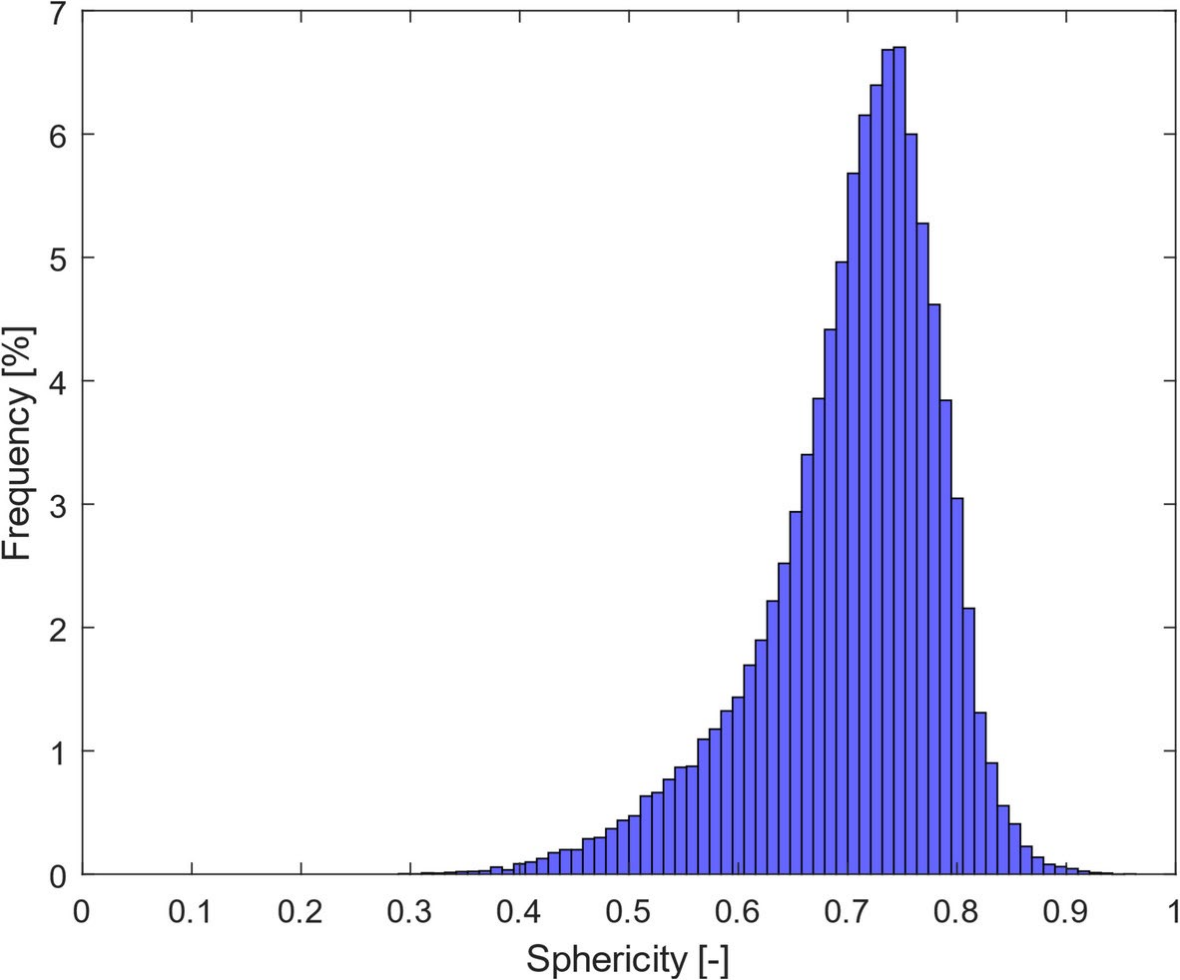
Distribution of sphericity values for the particles in MDP2.

Using the segmented images in Dragonfly and analysing the particles as the region of interest, the sphericity distribution for MDP2, as an example, could be obtained in the form of a histogram, displayed in Fig. 8. The shape of the particles deviated from sphere and the sphericity of particles for the samples used in this study was mainly distributed between around 0.6 and 0.8. Other samples also showed similar sphericity distribution.

#### Polydispersed samples

**Fig. 9:**
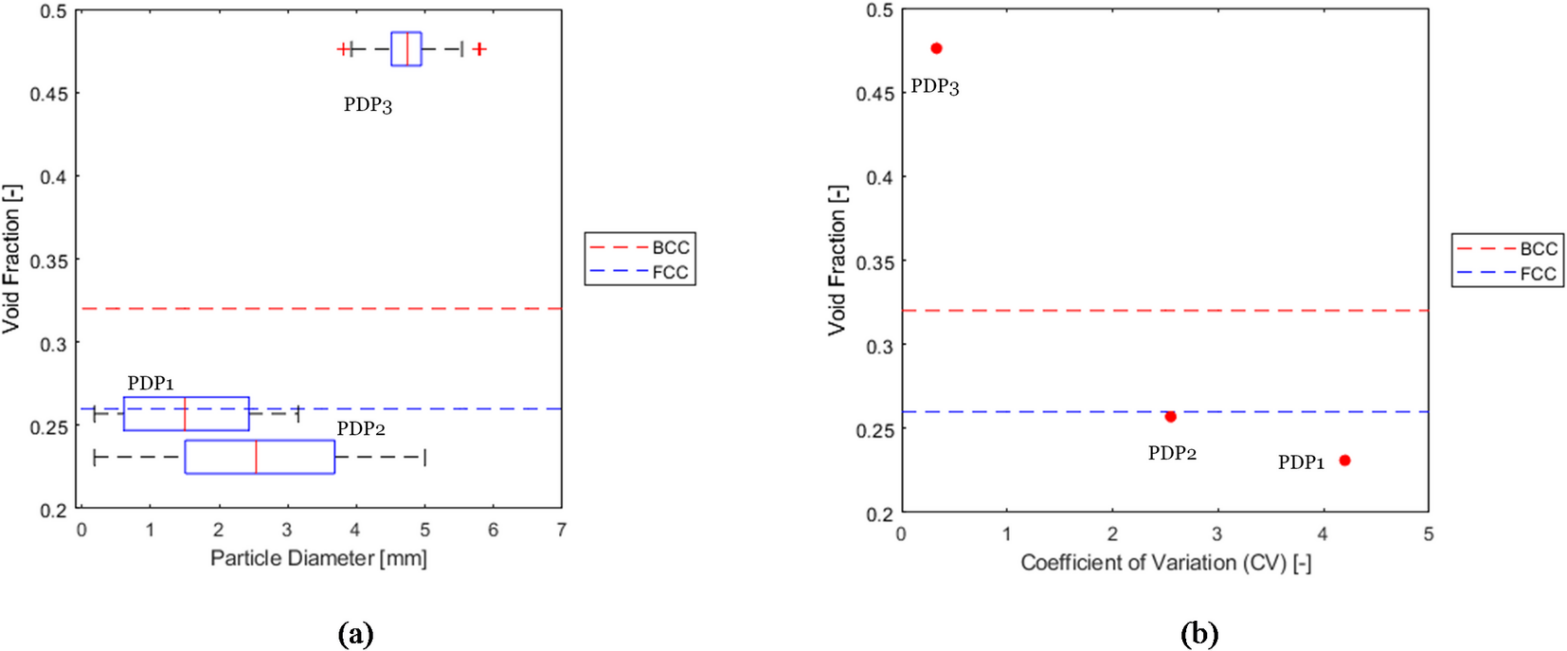
A comparison between the void fractions of samples PDP1, PDP2, and PDP3, and the analytical values for void fractions for BCC and FCC packing arrangements. (a) Function of Particle size, (b) function of CV.

As presented in Fig. 9a and b, PDP1 and PDP2 exhibited smaller volume-averaged void fractions than the minimum analytical value associated with FCC packings. This reduction in void fraction is due to smaller particles (0.18 mm) fitting into the spaces between larger particles (3 mm for PDP1 and 5 mm for PDP2), decreasing the void fraction compared to cases where all particles are mono-sized. In contrast, PDP3, which has a smaller coefficient of variation (*CV*), features a majority of particles of similar size (moderately dispersed as defined in 2.1.). Therefore, smallest particles are too large to fill the gaps between larger ones. Consequently, the void fraction for PDP3 was similar to that of monodispersed packings (0.47 for PDP3). This observation confirms that the coefficient of variation is an effective criterion for assessing the effect of polydispersity on void fraction distribution within packed beds of particles which is shown in Fig. 9b.

### Tortuosity distribution

#### Method validation

As mentioned previously, the tortuosity in packed beds is not a single value but a range of values derived from calculating all possible fluid pathways through the voids between particles. This tortuosity distribution provides valuable insights into the structure of the pore space within packed beds. In the following step, the ideal arrangement of SC (Simple Cubic) together with BCC and FCC have been generated in STAR-CCM + and then were imported into DRAGONFLY, following the procedure detailed in the image processing section. These ideal arrangements were chosen because they have well-defined tortuosity values in the literature, allowing for a direct comparison with the results obtained from the pore network models. The pore space structure of each bed was then extracted, and conventional PNM and dense graph were generated using the respective methods described in sections “Calculation of tortuosity based on the conventional pore network model (PNM)” and “Calculation of tortuosity based on the dense graph approach”. The tortuosity distribution of SC, BCC, and FCC packings was subsequently calculated and compared with the models in the literature, which are listed in Table 2. Table 2 provides a summary of these correlations along with their applicable void fraction range. The majority of these correlations are developed for spherical and monodispersed particles and are based on experimental data, such as diffusivity or conductivity measurements.

**Table 2:** Formulas for calculating tortuosity in packed beds of spherical particles in the literature.

Model Name	Correlation	Applicable void fraction range	Derivation method	Type of tortuosity
Maxwell (1873) ^14^	$\frac{3}{2} - \frac{1}{2}\varepsilon$	Not specified	Analytical	Electrical
Bartell & Osterhof (1928) ^15^	$\frac{\pi }{2}$	$\varepsilon =0.4$	Experimental	Hydraulic
Carman (1937) ^3^	$\sqrt 2$	$\varepsilon =0.4$	Experimental	Hydraulic
Weissberg (1963) ^16^	$1 - \frac{{{\text{ln}}\left( \varepsilon \right)}}{2}$	$0.36<\varepsilon <1$	Analytical	Diffusive
Bear (1972) ^17^	$\frac{1}{{{\varepsilon ^{0.4}}}}$	Not specified	Experimental	Geometric
Comiti & Renaud (1989) ^18^	$1 - 0.41{\text{ln}}\left( {\text{\varvec{\upvarepsilon}}} \right)$	Not specified	Experimental	Geometric
Du Plessis & Masliyah (1991) ^19^	$\frac{\varepsilon }{{1 - \sqrt[3]{{{{\left( {1 - \varepsilon } \right)}^2}}}}}$	Not specified	Analytical	Geometric
Iversen & Jorgensen (1993) ^20^	$\sqrt {1+2\left( {1 - \varepsilon } \right)}$	$0.4<\varepsilon <0.9$	Experimental	Diffusive
Boudreau (1996) ^21^	$\sqrt {1 - ln({\varepsilon ^2})}$	Not specified	Analytical	Diffusive
Ahmadi et al. (2011) ^22^	$\sqrt {\frac{{2\varepsilon }}{{3\left[ {1 - 1.209{{\left( {1 - \varepsilon } \right)}^{\frac{2}{3}}}} \right]}}+\frac{1}{3}}$	$\varepsilon >0.4$	Analytical	Geometric

**Fig. 10:**
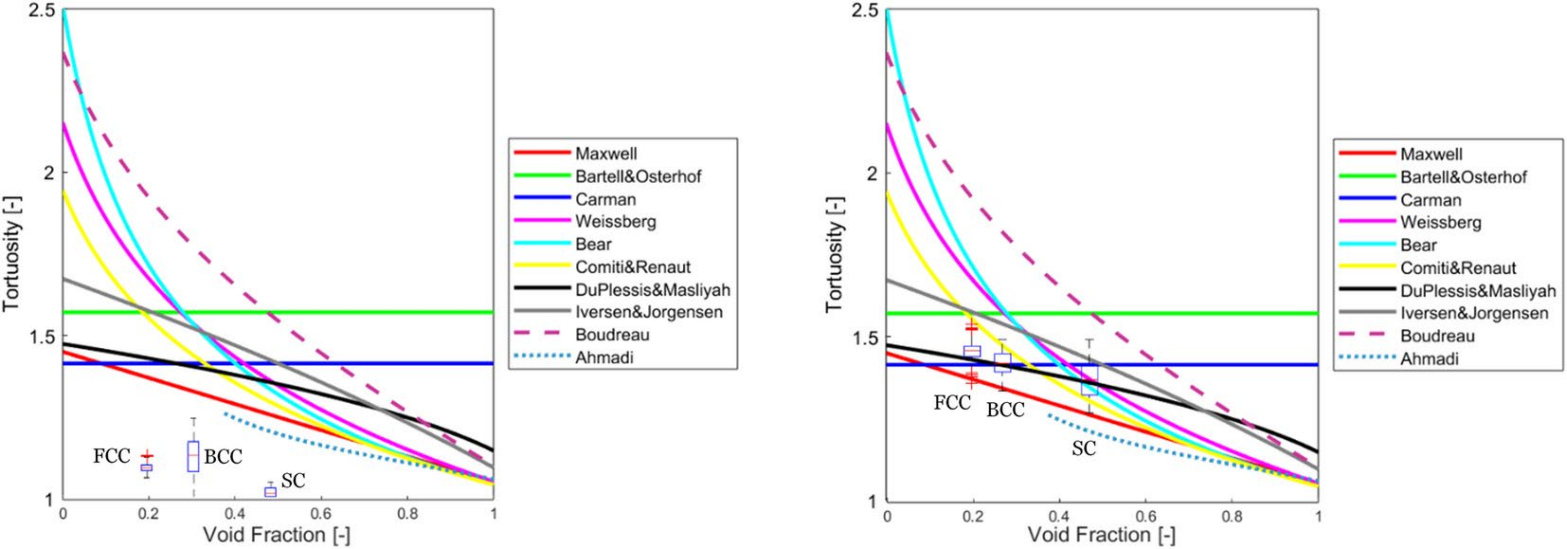
The tortuosity distribution of SC, BCC, and FCC packings compared to models in the literature (Table 2). The results were obtained by using conventional PNM (left) and dense graph (right).

As shown in the Fig. 10, the conventional PNM method underestimates the tortuosity distributions compared to all the models available in the literature and there exists not even one overlap in the range.

This is due to its simplified representation of the pore structure. In conventional PNM, the transport pathways between pores are assumed to be straight lines, neglecting the actual curved or complex trajectories present in a real porous medium. This simplification leads to an underestimation of the actual path lengths that fluids or particles must traverse, thereby reducing the calculated tortuosity values. However, the routes between pores are affected by the curves on the surface of particles.

In contrast, a dense graph representation accounts for the intricate connectivity and curved pathways between pores, providing a more realistic depiction of the porous structure. By incorporating these curved paths, the model more accurately captures the longer and more complex transport routes within the material, leading to a more precise estimation of tortuosity distributions. Thus, the underestimation in conventional PNM is primarily a result of its geometric simplifications, which do not fully capture the true complexity of the pore network. This could be especially important for irregularly-shaped samples because their pore space can be a lot more complicated than packed beds of regular shaped particles like spheres and cylinders due to their non-smooth surface.

The dense graph has better consistency with the models in the literature. Among the analytical models, the one presented by Du Plessis and Masliyah predicted the median tortuosity values with over 95% accuracy. This model, which was derived for isotropic granular media, does not account for the formation of isolated pore clusters that could obstruct continuous fluid flow. This omission seems to have resulted in a strong agreement between the model and the median values.

Another noteworthy observation from Fig. 10 is that the minimum tortuosity values from the dense graph overlap with those predicted by the Maxwell model. Maxwell proposed the formula listed in Table 2 for electrical tortuosity in a conducting medium with dilute, nonconducting spheres, where the void fractions approach one. For SC, BCC, and FCC packings, there could be at least one pathway connecting the inlet and outlet of the container with minimal particle barriers, leading to the minimum possible tortuosity value.

As the tortuosity distribution obtained by the dense graph is in better agreement with the models for the idealistic packings, further results in this study have been presented by the dense graph.

#### Monodispersed samples

**Fig. 11:**
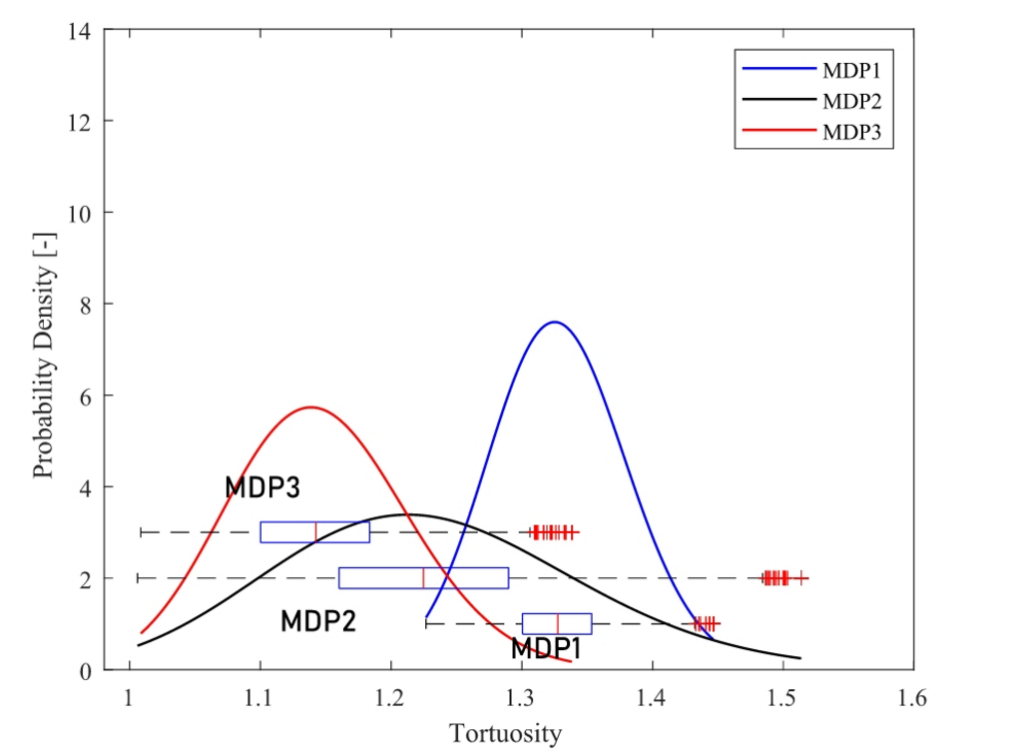
Distribution of the tortuosity within monodispersed samples visually compared with the box chart of tortuosity distribution by dense graph.

Figure 11 illustrates the tortuosity distribution for monodispersed samples using dense graph approach.

MDP1 exhibits higher tortuosity, followed by MDP2. Smaller particles with smaller void fractions showed higher tortuosity values. It could be related to the ratio of domain (sample holder) radius to particle radius as presented in Ref.^49^ for mono-sized spherical particles.

**Fig. 12:**
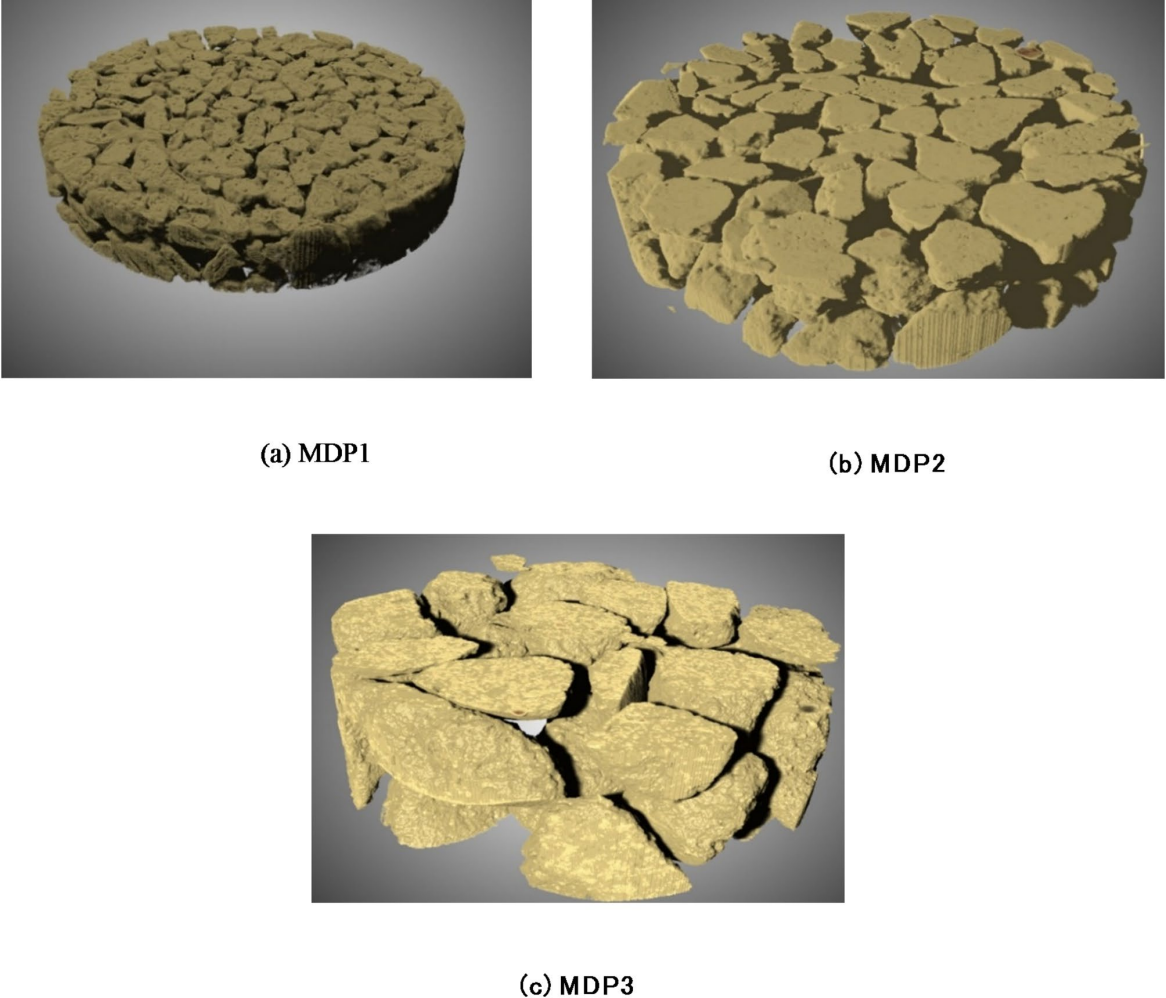
3D view of pack bed obtained from the XMT monodisperse pack beds (a) MDP1, (b) MDP2, (c) MDP3. The 3D image was generated using DRAGONFLY 2022.2 (software available at https://www.theobjects.com/dragonfly)^47^.

To have a better understanding of tortuosity distribution, the 3D views of three monodispersed packed beds obtained by XMT are presented in Fig. 12. Decreasing particle sizes in monodispersed samples adds additional bends to the pathways which leads to a higher mean values of tortuosity distribution. Especially, the bed from larger particles seems to lack the presence of small channels that can be represented as throats in PNMs. This may in turn result in the dominance of low tortuosity.

**Fig. 13:**
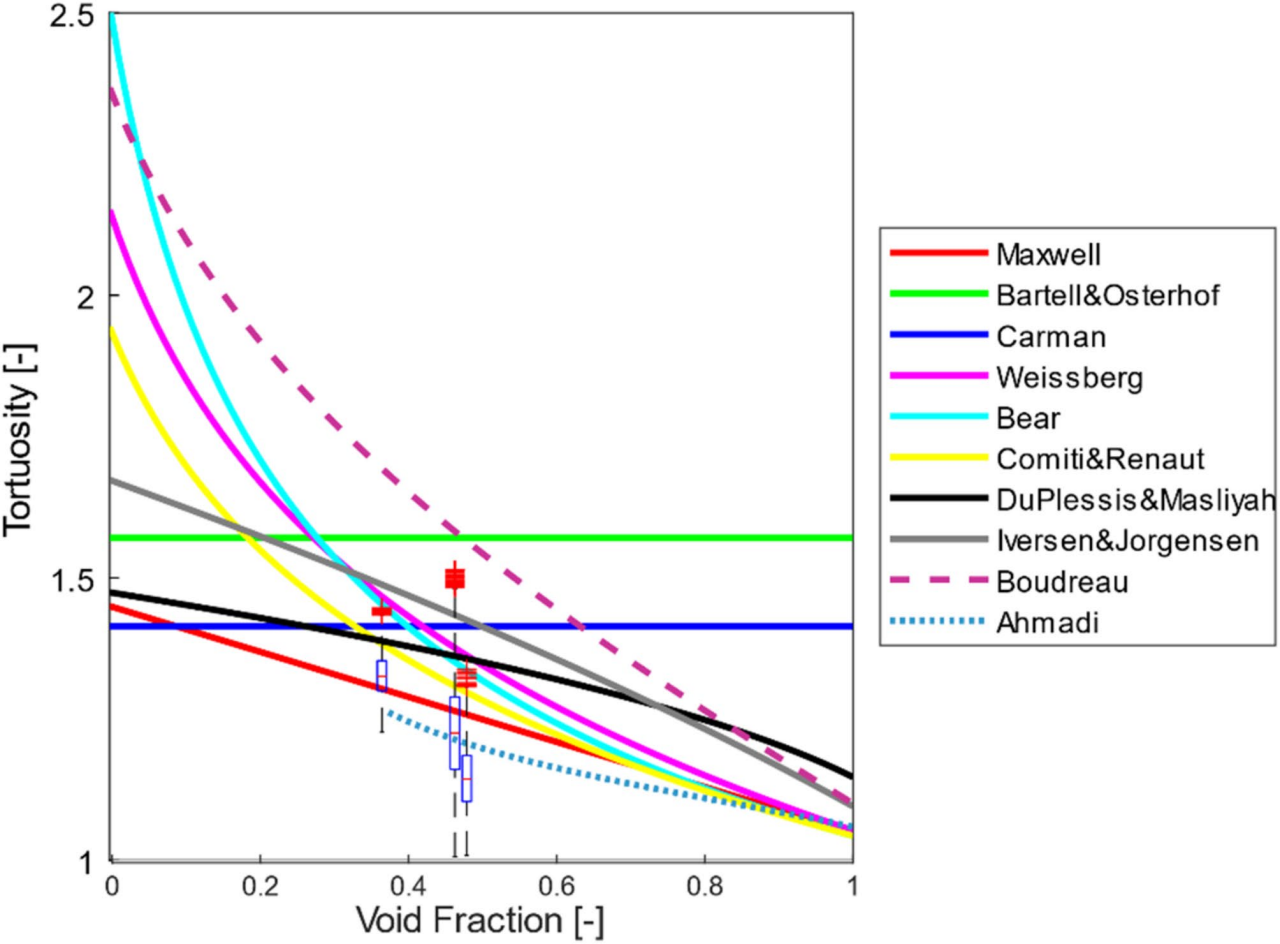
The tortuosity distributions of monodispersed particles (MDP samples) obtained from the dense graph approach for XMT-based images, in comparison with the models in literature (Table 2).

Figure 13 compares the tortuosity models in literature with the distribution span of tortuosity obtained from the dense graph approach. As shown in the figure, the tortuosity distributions obtained by the dense graph approach for all samples are within the range of the models in the literature and give a similar trend against void fraction. One can find at least one model in the literature that could describe part of the distributions. Hereby, the tortuosity values for the three monodispersed samples could be described by the analytical model of Ahmadi et al.^22^ and the deviation of the mean values from the model values is less than 2%. One reason for this agreement could be the fact that this analytical model is a modified correlation for tortuosity that adds the packing factor to the older literature values. The model also considered the packing of cubic and tetrahedral particles. This could help predict the packings of real-life irregularly-shaped particles more than the previous analytical models. The results also confirm that the dense graph is a useful approach for calculating tortuosity for non-spherical mono-sized particles. Finally, the tortuosity range is lower than the mean values predicted by Du Plessis and Masliyah model, which was the best fit with SC, BCC, and FCC packings (spherical monodispersed samples). It could be interpreted that the non-spherical particles have lower tortuosity values than the spherical particles. This observation is reasonable because at least one particle tends to have convex points at the contact points between non-spherical particles as shown in Fig. 12 and prevent the formation of large pore- blocking volumes nearby.

#### Polydispersed samples

**Fig. 14:**
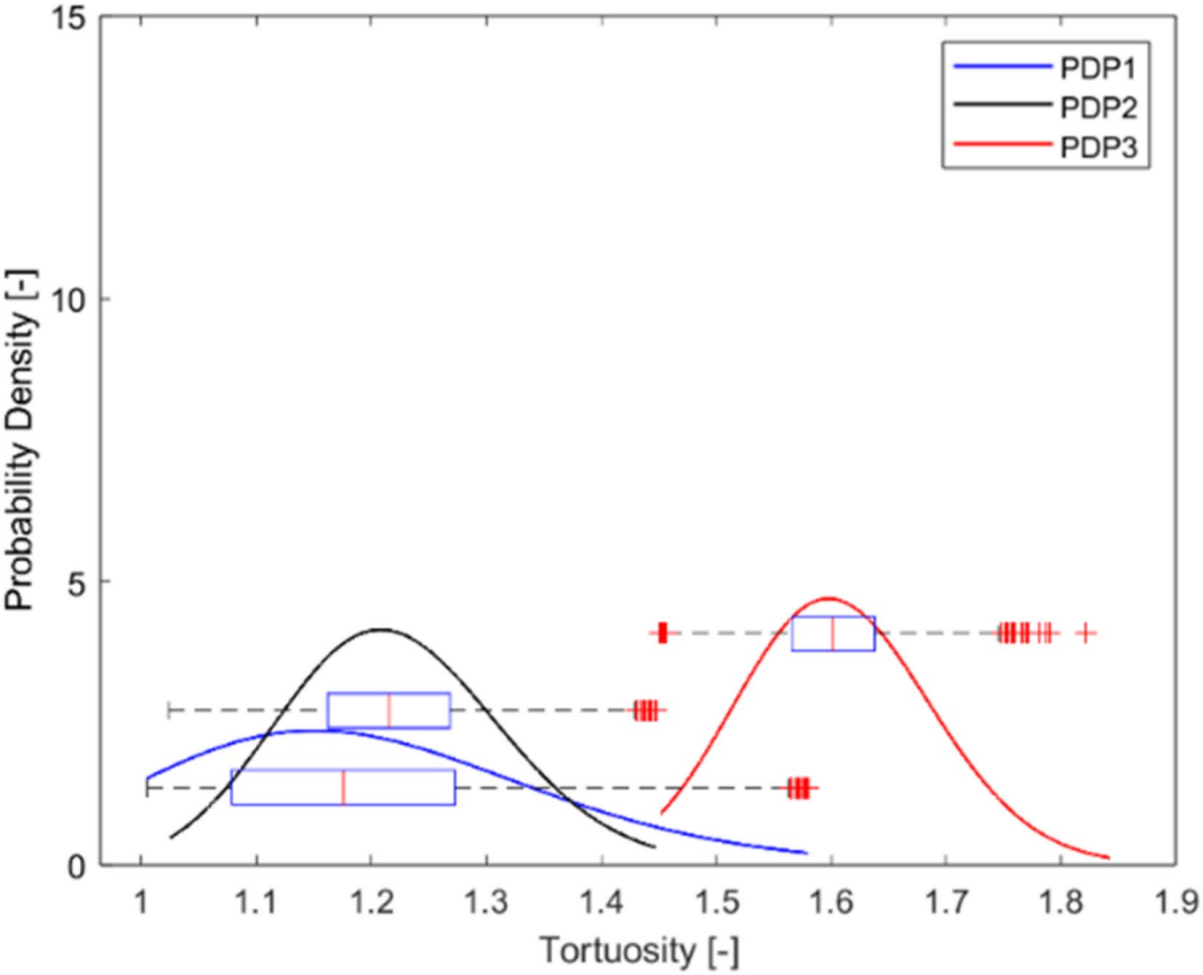
Log-normal distribution of tortuosity within polydispersed samples, visually compared with the box chart of tortuosity distribution obtained using dense graph.

Figure 14 presents the tortuosity distribution of three polydispersed samples (PDP1, PDP2, and PDP3) obtained by the dense graph method. The dense graph method shows slightly larger mean values for PDP3 and smaller mean values for PDP1 and PDP2.

The 3D views of three packed beds with polydispersed particles are presented in Fig. 12 for a better understanding of the tortuosity distribution. PDP3 is a moderately polydispersed packed bed while PDP1 and PDP2 are highly polydispersed as shown in Table 1 (*CV* values). In PDP1 and PDP2, the gaps between larger particles have been filled with smaller particles, resulting in a lower void fraction compared to all other samples. Moreover, the effect of void filling by smaller particles appears to make the channel narrower, but without blocking completely to increase the tortuosity significantly.

On the other hand, PDP3 is considered to be a moderately polydispersed bed and the behavior is closer to monodispersed samples rather than the polydispersed ones. Since the size differences among particles are relatively small, smaller particles cannot fit in the gaps between larger particles without displacing them. Such smaller, yet relatively large particles would add additional long bends surrounding their perimeters at the original gaps where fluid could have otherwise passed through in a straight line. This may result in higher tortuosity than monodispersed particles, in contrast with highly polydispersed particles (PDP1 and PDP2).

**Fig. 15:**
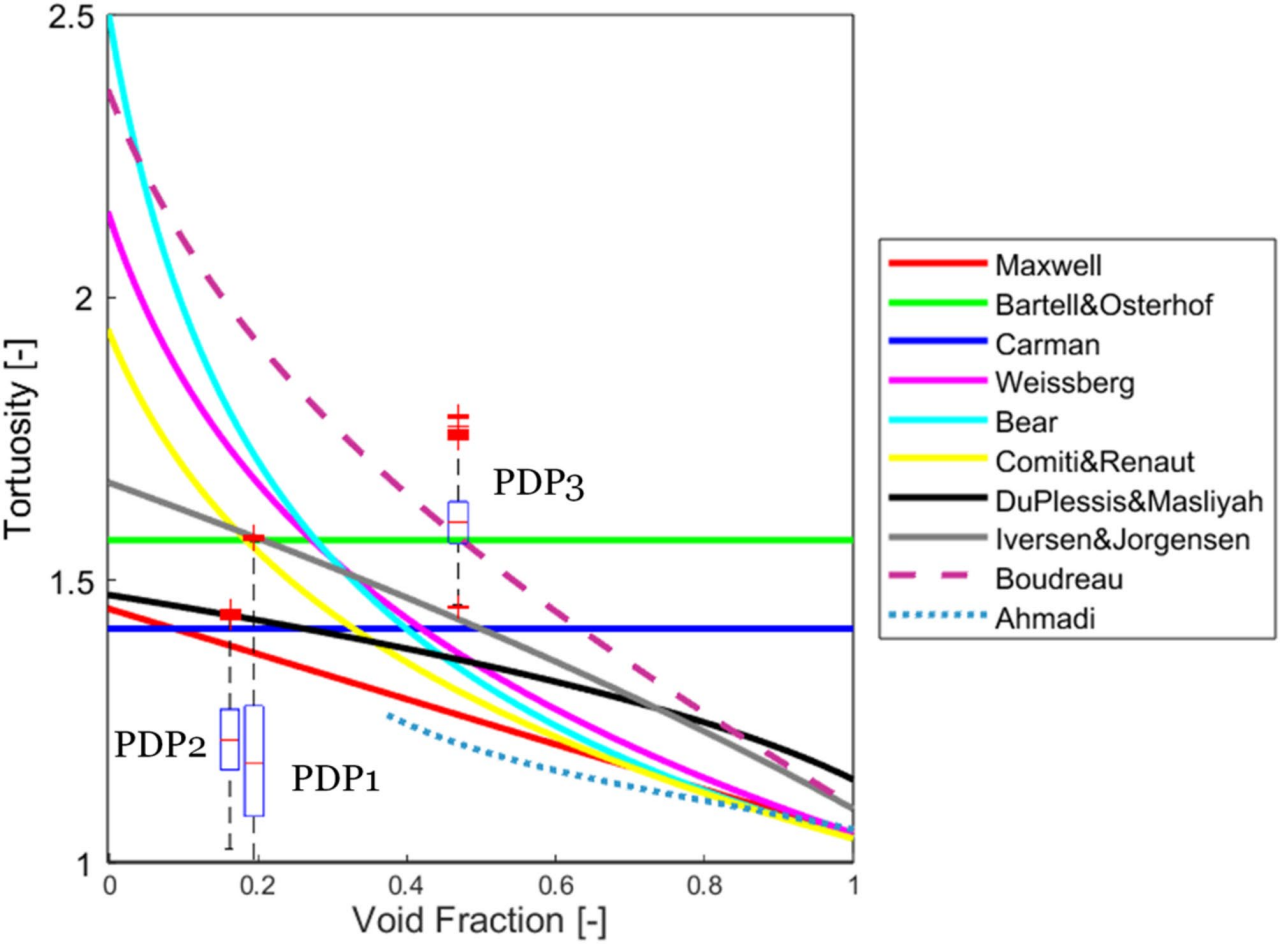
The tortuosity distributions of polydispersed particles (PDP samples) obtained from the dense graph approach for XMT-based images, in comparison with the models in literature (Table 2).

Figure 15 illustrates the tortuosity distributions of the polydispersed samples in comparison with the models in the literature. The literature models overpredicted the tortuosity values for PDP1 and PDP2. This deviation highlights the fact that empirical models mostly consider spherical monodispersed packed beds and cannot predict the effect of polydispersity, especially when the degree of polydispersity is high. Meanwhile, the values from several empirical models overlapped those of PDP3. The reason for the difference between PDP3 and the other two polydispersed samples could be related to the differences in the degree of polydispersity, represented by the coefficient of variation (*CV*). As it was mentioned before, PDP1 and PDP2 are highly polydispersed (*CV* > > 0.3) and PDP3 is moderately polydispersed and on the threshold between mono and polydispersity (*CV* = 0.33).

**Fig. 16:**
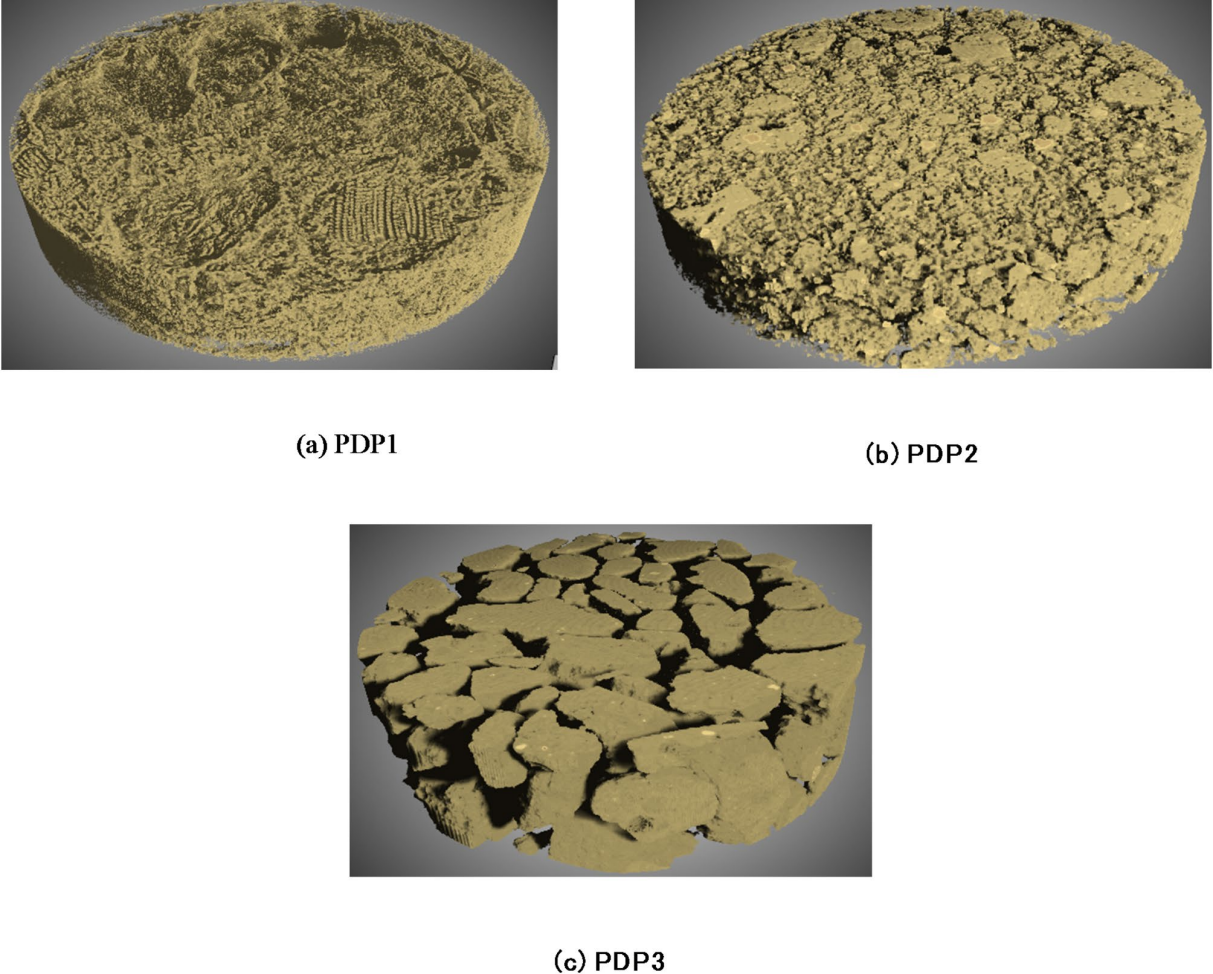
XMT-based 3D view of monodispersed packed beds for (a) PDP1, (b) PDP2, and (c) PDP3. The 3D image was generated using DRAGONFLY 2022.2 (software available at https://www.theobjects.com/dragonfly)^47^.

Even though the results of monodispersed and polydispersed samples cannot be directly compared (because of different ranges of void fraction), the tortuosity of PDP1 and PDP2 seems to be generally smaller than the monodispersed samples. This is because the void fractions decreased by small particles filling the big voids between large particles, and this did not add additional large bends to the pathways (see Fig. 16). However, a more comprehensive investigation is needed to draw further conclusions.

## Conclusions

This study highlights the significant influence of particle shape and particle size distribution on packed bed void fraction and tortuosity. Within the monodispersed samples, an increase in particle diameter correlates with a higher void fraction, deviating from the analytical value for mono-sized spherical packing. This deviation is primarily related to the ratio of domain (sample holder) radius to particle radius. Furthermore, the study reveals that the degree of polydispersity, measured by the coefficient of variation (*CV*), significantly impacts void fraction. Higher *CV* (i.e. more polydispersed sample) was associated with lower void fractions because smaller particles are generally smaller than larger particles in the distribution and can fill the void between larger particles.

Additionally, the study employed two methods to extract pore-throat network from the void space structure of the porous packed beds. The conventional PNM with cylindrical throats approximation and the dense graph approach were compared with the estimation of tortuosity distributions within the idealistic packings of BCC (body-centered cubic) and FCC (face-centered cubic). It was demonstrated that the dense graph method yields values close to models in the literature.

Furthermore, it was shown that tortuosity is a function of void fraction in monodispersed samples, where decreasing void fraction led to an increase in tortuosity. Existing models in the literature could successfully predict the tortuosity of the packed bed with non-spherical particles (with the sphericity of 0.6–0.8) in this study. Additionally, highly polydispersed beds exhibit tortuosity distributions that are similar to those of monodispersed beds with the particle size similar to the largest end of the distribution, despite having much lower void fraction. The visualization of the bed showed that this is because small particles fill the voids between large particles without completely blocking the passage.

Although PNM was chosen as the most suitable method for this study, we acknowledge the limitations of geometrical tortuosity in estimating thermophysical parameters, as discussed in the manuscript. In future work, we will explore direct tortuosity estimation techniques to complement our findings and address these limitations.

## Data Availability

The data for this study is available upon request by the corresponding author (contact: zahra.ghasemi.monfared@associated.ltu.se).

## List of symbols

$A_{section}$: Circular section’s area [m^2^]
$A_{void}$: Section’s area of void space [m^2^]
*A*_*p*_: Surface area of the particle [m^2^]
*CV*: Coefficient of variation [-]
$$d_{p}$$: Particle size [m]
$$D_{A}$$: Molecular diffusivity [m^2^/s]
$$D_{eff}$$: Effective diffusivity [m^2^/s]
$$D_{Kn}$$: Knudsen diffusivity [m^2^/s]
*D*_*max*_: Maximum particle sizes [mm]
*D*_*min*_: Minimum particle sizes [mm]
*R*: Radius of the cylinder [m]
r: Radius of the subdomain [m]
*V*_*p*_: Volume of the particle [m^3^]

## Greek letters

$\varepsilon$: The bed void fraction [-]
$\overline{\varepsilon }_{section}$: Area-averaged void fraction [-]
$$k$$: Permeability [m^2^]
$$\tau$$: Tortuosity [-]
σ: Logarithmic standard deviation in log-normal distribution [-]
$\Phi_{s}$: Sphericity of the particles [-]
